# Prediction of emergence from the minimally conscious state at 6 months using acute-phase clinical and event-related potential indicators in patients with prolonged disorders of consciousness: a single-center retrospective cohort study

**DOI:** 10.3389/fneur.2026.1948269

**Published:** 2026-10-28

**Authors:** Shenghua Cui, Ping Chen, Jinjin Li, Shuai Cheng, Chenyu Liu, Haiyang Bian, Zhenyu Nie, Yizhao Chen

**Affiliations:** 1 Department of Neurosurgery, Zhujiang Hospital of Southern Medical University, The National Key Clinical Specialty, The Neurosurgery Institute of Guangdong Province, Guangdong Provincial Key Laboratory on Brain Function Repair and Regeneration, The Engineering Technology Research Center of Education Ministry of China, Southern Medical University, Guangzhou, China; 2 The Second Affiliated Hospital, Department of Neuromedicine Center, Hengyang Medical School, University of South China, Hengyang, Hunan, China; 3 Department of Neurosurgery, The Seventh Affiliated Hospital, Southern Medical University, Foshan, China

**Keywords:** acute phase, event-related potential, multimodal indicators, prediction model, prolonged disorders of consciousness

## Abstract

**Objective:**

To identify acute-phase predictors of long-term outcome in patients with disorders of consciousness (DoC) and establish a multimodal prediction model.

**Methods:**

This retrospective study enrolled 227 patients with prolonged DoC at the Second Affiliated Hospital of the University of South China between August 2019 and December 2025. The primary endpoint was emergence from the minimally conscious state (eMCS) at 6 months after onset, defined as functional communication or functional use of objects on the Coma Recovery Scale-Revised (CRS-R), which was assessed at 1, 3 and 6 months after onset. Clinical, imaging and neurophysiological acute-phase data (≤28 days after onset) were collected. Variables significant in univariate analysis entered multivariable logistic regression; clinical, electrophysiological and combined models were compared by the area under the receiver operating characteristic curve (AUC), calibration and clinical utility, and the final model was presented as a nomogram.

**Results:**

Univariate screening identified age, acute-phase FOUR score, MMN and P300 amplitudes, cerebral herniation, etiology, hydrocephalus and st-SCS surgery (all *p* < 0.05); age, etiology, FOUR score and Fz P300 amplitude remained independent in multivariable analysis. Discrimination was acceptable for the clinical (AUC = 0.745, 95% CI 0.681–0.809) and multimodal (AUC = 0.772, 95% CI 0.711–0.832) models, but not significantly different (ΔAUC = 0.027, DeLong test *p* = 0.115); after bootstrap correction the multimodal AUC was 0.735, with adequate calibration (Hosmer-Lemeshow *p* = 0.634). Adding ERP measures improved reclassification relative to the clinical model (continuous NRI = 0.317, 95% CI 0.062–0.572, *p* = 0.015; IDI = 0.039, 0.014–0.065, *p* = 0.003). Performance did not differ between TBI and non-TBI patients (ΔAUC = 0.061, *p* = 0.346), although these comparisons were underpowered.

**Conclusion:**

Acute-phase FOUR score, age, etiology and the Fz P300 amplitude were independently associated with emergence from the minimally conscious state at 6 months in prolonged DoC. A nine-indicator multimodal model showed acceptable, internally validated discrimination (AUC = 0.772; bootstrap-corrected 0.735) and modestly improved reclassification over clinical variables alone (continuous NRI = 0.317; IDI = 0.039). External validation is required before clinical use.

## Introduction

Severe brain injury from various causes, including traumatic brain injury, cerebrovascular accidents, and hypoxic–ischemic encephalopathy, frequently leads to disorders of consciousness (DoC). When the condition persists beyond 28 days, it is termed prolonged DoC (pDoC), which encompasses the vegetative state (VS) and the minimally conscious state (MCS) (1–3).

In China, an estimated 70,000 to 100,000 new pDoC cases emerge annually. They place substantial emotional and economic burdens on families and society (4). Early prediction of whether a DoC patient will regain consciousness can guide clinicians toward more rational treatment decisions. It also helps families make timely medical choices and facilitates equitable allocation of healthcare resources.

Currently, prognostic assessment for DoC patients in clinical practice relies heavily on physicians’ clinical observation and personal experience. This approach is inherently subjective and susceptible to confounding factors (5). The academic community has been actively exploring early prediction models across multiple modalities, including clinical characteristics, neuroelectrophysiology, neuroimaging, and physiological or biochemical markers. However, single-modality predictors each carry technical limitations, resulting in suboptimal accuracy, sensitivity, and specificity (6–10). Integrating multiple techniques is therefore necessary to improve prognostic accuracy in DoC patients.

In this study, we retrospectively collected clinical characteristics, imaging findings, and neurophysiological indicators from the acute phase (in the first month after onset) of pDoC patients. Through univariate and multivariate regression analyses, we identified independent predictors of a favorable outcome, compared the predictive performance of single-modality versus multimodal approaches, and developed models for predicting emergence from the minimally conscious state at 6 months. Discrimination (including internal validation), calibration and clinical utility were assessed, and the terminology and outcome definition used throughout the revised manuscript follow the current diagnostic criteria for DoC. To our knowledge, no previous study has addressed early prediction of long-term outcomes using acute-phase multimodal data in this way.

## Materials and methods

### Study subjects

This retrospective study included pDoC patients admitted to the Department of Neurosurgery at the Second Affiliated Hospital of the University of South China between August 2019 and December 2025. During this period, ERP recordings were obtained in 436 patients with severe brain injury; the examination was part of the routine assessment of every patient with DoC admitted to the unit rather than being requested selectively, and these patients were identified from the consecutive records of examinations stored on the event-related potential system used for all recordings (WJ-1A, Guangzhou Runjie Medical Instrument Co., Ltd.). After exclusion of 209 patients, 227 patients were analyzed (Figure 1). The reasons for exclusion were an ERP examination outside the 1-month post-onset window (*n* = 183), failure to meet the inclusion criteria (*n* = 19), loss to follow-up or incomplete 6-month data (*n* = 6) and other reasons (*n* = 1). An ERP recording in which an MMN or a P300 could be identified was required for inclusion, and patients in whom no such component could be identified are counted among those who did not meet the inclusion criteria (*n* = 19); an identifiable component was recorded in each of the 227 analyzed patients.

**Figure 1:**
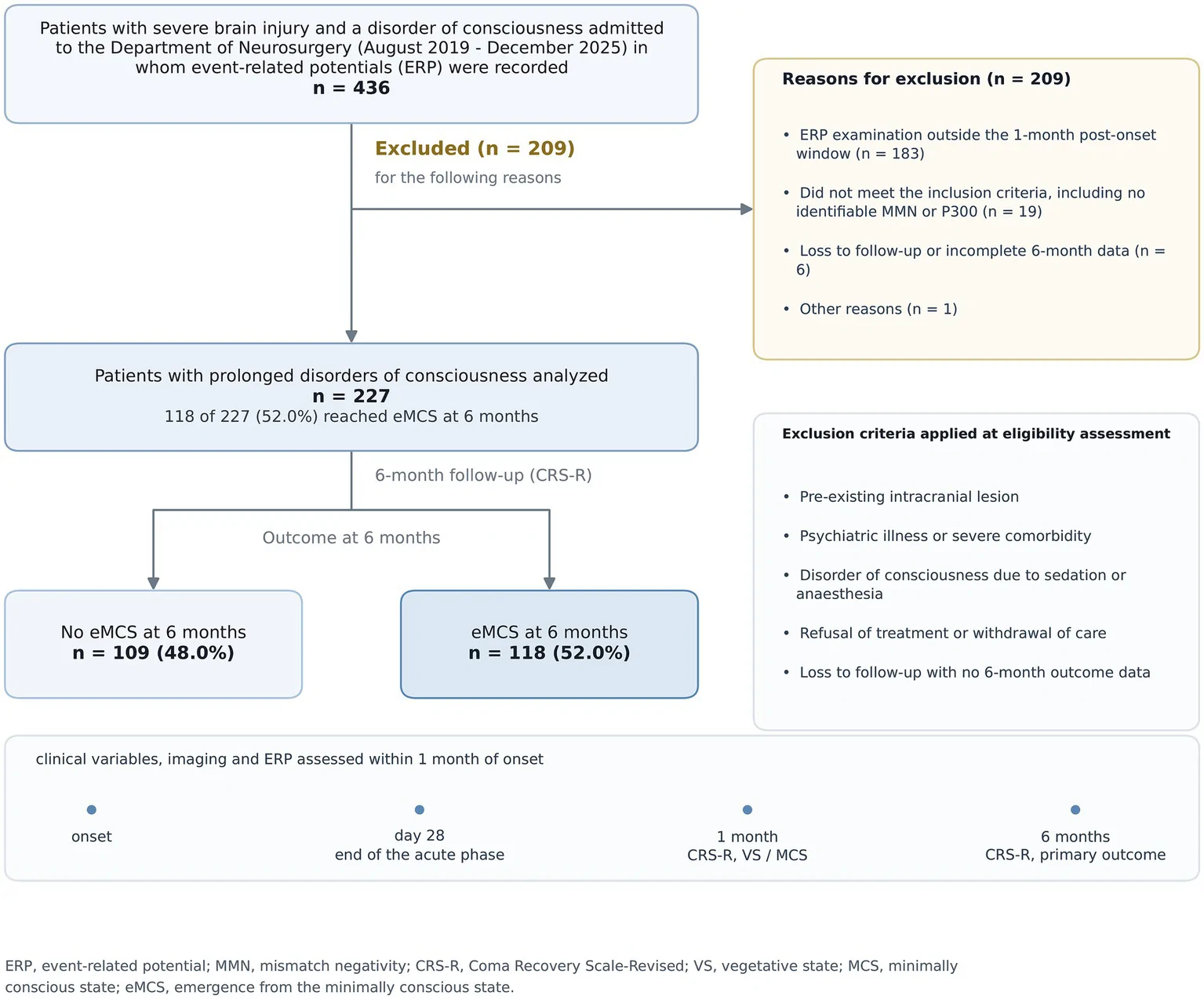
Flow chart of patient selection. Of 436 patients with severe brain injury in whom event-related potentials were recorded, 209 patients were excluded and 227 patients were analyzed. The reasons for exclusion, in the order in which they were applied, were an ERP examination outside the 1-month post-onset window (*n* = 183), failure to meet the inclusion criteria (*n* = 19; this category includes patients in whom no MMN or P300 could be identified), loss to follow-up or incomplete 6-month data (*n* = 6) and other reasons (*n* = 1).

Inclusion criteria were: (1) age 3–90 years; (2) a disorder of consciousness (coma, vegetative state or minimally conscious state) documented with the CRS-R at 1 month post-onset; patients who had already reached eMCS at that assessment were retained in the primary analysis (*n* = 28) and were excluded in a sensitivity analysis (Supplementary Table S3); (3) documented history of brain injury; (4) cranial CT or MRI confirming traumatic brain injury, cerebrovascular accident, or hypoxic–ischemic encephalopathy, with etiology recorded in the registry as one of these three categories, the subtype of cerebrovascular accident (ischemic or hemorrhagic) not being recorded separately; (5) ERP examination performed within 1 month of onset, yielding a recording in which an MMN or a P300 could be identified; (6) a consciousness level documented at the 6-month assessment or, in patients who died before that assessment, at the last assessment before death. Patients who regained consciousness before the 1-month assessment, and therefore never met the criteria for a prolonged disorder of consciousness, did not satisfy criterion (2) and were not part of the 227 patients analyzed.

Exclusion criteria were: (1) pre-existing intracranial lesions or history of psychiatric illness; (2) DoC secondary to sedation or anesthesia; (3) severe pre-existing comorbidities (e.g., coronary heart disease, renal failure); (4) refusal of standard treatment or withdrawal of care; (5) loss to follow-up with no information on the 6-month outcome. Patients who died before the 6-month assessment were retained in the analysis; death was not counted as recovery of consciousness, and the last recorded consciousness level was used for the 6-month time point. The Glasgow Outcome Scale (GOS) score is recorded on the day on which the patient completes six months from onset, whereas the consciousness level recorded for the 6-month time point is the highest level reached by that time; the two therefore describe different time points and need not agree in patients who died between assessments. Of the 46 patients with a GOS score of 1 (death) at 6 months, 45 were classified as not having achieved eMCS and one as having achieved it; 7 of the 46 had a CRS-R total score of 0 at every assessment, and all 7 were classified as coma (see Limitations).

This study adhered to the ethical principles of the latest Declaration of Helsinki and was approved by the Ethics Committee of the Second Affiliated Hospital of the University of South China (Approval No. 2025029). The requirement for informed consent was waived.

### Study design and data collection

Patients were classified according to whether eMCS had been achieved by the 6-month assessment (eMCS achieved vs. eMCS not achieved). eMCS was defined by the CRS-R criteria, that is, the presence of functional communication or functional use of objects, and not by a CRS-R total score threshold. Consciousness level (coma, vegetative state [VS], minimally conscious state minus [MCS-], minimally conscious state plus [MCS+], eMCS) and the CRS-R total score were recorded at 1, 3 and 6 months after onset. The primary outcome was the consciousness level at 6 months. The following acute-phase (≤28 days after onset) data were collected: demographic characteristics (sex, age), clinical features (etiology [TBI vs. non-TBI], cerebral herniation, skull defect, hydrocephalus, st-SCS surgery, admission FOUR score), neuroimaging (Rotterdam CT score and, when available, MRI), and neurophysiological indicators (MMN and P300 components from ERP, including latency and amplitude at Fz and Cz channels). Etiology was recorded in the registry as one of three categories (traumatic brain injury, cerebrovascular accident or hypoxic–ischemic encephalopathy) and was analyzed as TBI versus non-TBI; the cerebrovascular accident category comprised both ischemic and hemorrhagic presentations, and the subtype was not recorded separately. Hypoxic–ischemic encephalopathy was defined as an acute hypoxic–ischemic event of any mechanism (in this cohort cardiac arrest, drowning, carbon monoxide poisoning and status epilepticus) with clinical and neuroimaging findings consistent with hypoxic–ischemic brain injury; documented cardiac arrest was not required, and 9 of the 11 patients with this etiology had a documented cardiac arrest (in one of them after drowning) whereas 2 did not (one had carbon monoxide poisoning and one status epilepticus). The interval between injury and ERP recording (days_erp) was also recorded. Functional status at 6 months was assessed with the Glasgow Outcome Scale (GOS) and is reported as a secondary descriptive outcome; GOS was not used as the primary outcome, because it describes general disability rather than consciousness.

### Event-related potential acquisition

ERP recordings were obtained at the bedside in the neurosurgical intensive care unit, with the patient lying supine in a quiet environment. No sedation was administered for the recordings, which were performed with the patient awake and with the eyes open. Signals were acquired with a WJ-1A medical event-related potential system (Guangzhou Runjie Medical Instrument Co., Ltd., Guangzhou, China). Electrodes mounted in an electroencephalographic cap were placed at Fz, Cz, F3 and F4 according to the international 10–20 system, with conductive paste injected at each site, and were referenced to linked earlobe electrodes (A1, A2); the ground electrode was placed at Fpz, and electrode impedance was kept below 30 kΩ. Two independent stimulus modules were used. For the mismatch negativity (MMN), a classic auditory oddball paradigm was applied, in which standard tones (1,000 Hz) and deviant tones (1,200 Hz) were presented in a 9:1 ratio, with an averaging epoch of 400 ms and an inter-stimulus interval of 500 ms. For the P300, a three-stimulus auditory oddball paradigm was applied, in which standard, deviant and novel stimuli were presented in an 8:1:1 ratio, with an averaging epoch of 500 ms and an inter-stimulus interval of 1,000 ms. Tones of 75 ms duration were delivered binaurally through insert earphones at the presentation level set in the stimulus module of the recording system. The signal was band-pass filtered between 0.1 Hz and 25 Hz and sampled by the amplifier of the recording system; at least 50 accepted trials were averaged for each stimulus type, time-locked to stimulus onset and measured from the pre-stimulus baseline of the averaging epoch defined by the acquisition software. The difference waveform was obtained by subtracting the standard-stimulus ERP from the deviant-stimulus ERP. MMN was defined as the maximum negative deflection of the difference waveform within 100–250 ms, and P300 as the maximum positive deflection within 300 ± 50 ms; amplitude (μV) and latency (ms) were measured at Fz and Cz. MMN amplitude is negative, and the values are used as recorded, with the sign retained, so that a more negative value denotes a larger MMN amplitude. Latency was defined as the interval from stimulus onset to the maximum deflection within the corresponding window. Parameters were named by channel, component and parameter type, for example Fz_MMN_L for MMN latency at Fz and Fz_P300_A for P300 amplitude at Fz. Recordings from F3 and F4 were acquired, but only Fz and Cz were analyzed. Component latencies and amplitudes were taken from the analysis output generated by the recording system, which identifies the peak within the predefined window automatically for each channel; the values were therefore produced by the acquisition software rather than by subjective visual rating, and no assessor-blinding procedure was required. ERP recording and analysis were performed as part of routine clinical care.

### Behavioral assessment

Level of consciousness was assessed with the Chinese version of the Coma Recovery Scale-Revised (CRS-R) at 1, 3 and 6 months after onset. Assessments were performed at the bedside by trained neurosurgeons. At each time point the CRS-R was administered on three separate occasions, and the best of the three scores was used (5). Children aged 6 years or younger were assessed with the Coma Recovery Scale for Pediatrics (CRS-P) (11), the pediatric modification of the CRS-R, and the same behavioral framework was applied. Emergence from the MCS (eMCS) was defined as functional communication or functional use of objects on the CRS-R, in line with current consensus criteria, and not by a CRS-R total score. Consciousness levels were coded as coma, VS, MCS-, MCS + and eMCS, and the CRS-R total score was recorded at each time point. The lowest category is reported under the label used in the registry (coma); when it is recorded months after injury this denotes persistent unresponsiveness rather than acute coma (see Limitations). In the analyzed cohort the CRS-R total score at 6 months was 8 (4–12) in patients who did not reach eMCS and 23 (22–23) in those who did; 37 of the 118 patients who reached eMCS (31.4%) had a CRS-R total score below 23 (range 16–22), which illustrates why a total score threshold is not equivalent to the diagnosis of eMCS (Supplementary Table S6).

### Standard care

During the acute phase all patients received standard neurocritical care according to institutional protocols, comprising airway and ventilatory management including tracheostomy when required, enteral nutrition, thromboprophylaxis, seizure control, treatment of intracranial hypertension and of infection, early rehabilitation (positioning, passive range-of-motion exercises and tracheostomy care), and management of spasticity and dysautonomia. In addition to these measures, hyperbaric oxygen therapy, neurotrophic agents and limb function rehabilitation were provided routinely in the unit and were available to all patients throughout the study period; no other adjunctive therapy, for example therapeutic hypothermia, was applied routinely, and none of these treatments was allocated according to treatment group. Multidisciplinary follow-up was provided by neurosurgeons, rehabilitation physicians and therapists at the scheduled 1-, 3- and 6-month assessments, which were performed at outpatient visits, on the ward during an admission or, when the patient was unable to attend, by structured telephone follow-up with the principal caregiver. Short-term spinal cord stimulation (st-SCS) was offered to a subset of patients after discussion with the family. The indications and the stimulation protocol were those described in our previous reports (12, 13): a percutaneous epidural electrode inserted by puncture at the T7/T8 level with the tip advanced to the C2 level under fluoroscopic guidance, a pulse width of 120 μs, a stimulation frequency of 5 or 70 Hz selected according to the individual response (12), and an amplitude titrated between 1.0 and 3.0 V so as not to induce limb movement; the electrode was removed after two weeks of stimulation. No other neuromodulation (transcranial direct current stimulation, repetitive transcranial magnetic stimulation, deep brain stimulation or vagus nerve stimulation) was performed at any time during the study period. Other management decisions, including discussions about goals of care, were made by the treating clinicians independently of the prediction model, which was developed retrospectively and was not available to the treating team.

### Statistical analysis

All statistical analyses were performed using R software (version 4.4.2; R Foundation for Statistical Computing, Vienna, Austria) with the pROC, rms, PredictABEL and mice packages. Categorical variables are presented as counts (percentages) and compared using the chi-squared or Fisher exact test; continuous variables are presented as mean ± SD or median (interquartile range) and compared using the independent-samples *t* test or the Mann–Whitney U test, as appropriate.

Model specification. Nine acute-phase variables were significant in univariate analysis and were treated as candidate predictors: age, FOUR score, etiology, cerebral herniation, hydrocephalus, st-SCS surgery, Fz_MMN_A, Cz_MMN_A and Fz_P300_A. Three models were specified *a priori* by the domain of the predictors, without any data-driven variable selection: (i) a clinical model containing the six clinical variables (Age, FOUR, Etiology, Herniation, Hydrocephalus, SCS_Surgery); (ii) an electrophysiological model containing the three ERP amplitude measures (Fz_MMN_A, Cz_MMN_A, Fz_P300_A); and (iii) a multimodal model containing all nine candidate predictors, that is, the union of the clinical and electrophysiological variable sets. In parallel, the independent contribution of each candidate predictor was examined with a multivariable binary logistic regression that contained all nine candidate predictors. In that model, age (*p* = 0.007), FOUR score (*p* < 0.001), etiology (*p* = 0.016) and Fz_P300_A (*p* = 0.012) were significantly associated with the outcome, whereas cerebral herniation, hydrocephalus, st-SCS surgery, Fz_MMN_A and Cz_MMN_A were not (Supplementary Table S1). This nine-variable model describes the independent contribution of individual variables, whereas discrimination, calibration, reclassification and internal validation refer to the three *a priori* models, which contain no selected variables and are therefore not affected by selection instability. The multimodal model contained nine parameters for 118 events (13.1 events per variable), which exceeds the conventional minimum of 10 events per variable.

Discrimination, calibration and internal validation. Discrimination was assessed with the area under the receiver operating characteristic curve (AUC) and 95% confidence intervals (CI), and models were compared with the DeLong test for correlated curves and with the likelihood-ratio test where nested models were compared. Calibration was assessed with calibration plots (apparent and bootstrap-corrected), the calibration slope and intercept, the Brier score (with the scaled Brier score as an R2 analogue) and the Hosmer-Lemeshow goodness-of-fit test (8 groups). Internal validation used 1,000 bootstrap resamples and repeated 10-fold cross-validation, and the optimism-corrected AUC, calibration slope and Brier score are reported. The independent contribution of each variable in the nine-variable model is presented as a nomogram.

Clinical utility and reclassification. Clinical utility was evaluated with decision curve analysis, in which the net benefit of each model was compared with the two reference strategies ‘treat all’ and ‘treat none’ across threshold probabilities from 5 to 90%. The clinical value of adding electrophysiological information was further quantified by risk reclassification, using the clinical model as the reference model: the continuous net reclassification improvement (NRI) and the integrated discrimination improvement (IDI) were calculated with 1,000 bootstrap resamples, and the categorical NRI was calculated with risk strata of < 0.2, 0.2–0.5 and > 0.5. Because the continuous NRI is sensitive to the direction of reclassification and does not in itself demonstrate improved accuracy, its events and non-events components are reported separately.

Missing data. Within the analyzed cohort, ERP amplitude values were missing in 7 patients (3.1%; Fz_P300_A in 5 and Fz_MMN_A in 2). In each of these 7 patients the other component was recorded, so every patient in the analyzed cohort had at least one identifiable cortical response. Missing values were imputed by multiple imputation by chained equations using predictive mean matching for continuous variables and logistic regression for categorical variables (five imputations, 10 iterations), and the imputed datasets were pooled before analysis. A complete-case sensitivity analysis restricted to patients with both ERP latency and amplitude values available was also performed.

Sensitivity and subgroup analyses. Pre-specified sensitivity analyses repeated the multimodal model after: (i) adjustment for the interval between injury and ERP recording and restriction to ERP performed within 28 days of onset; (ii) restriction to ERP recorded 2–14 days or 15–31 days after onset; (iii) restriction to adults (≥18 years); (iv) exclusion of patients who died before the 6-month assessment (GOS 1); (v) exclusion of patients who had already reached eMCS at the 1-month assessment, and restriction to patients in VS or MCS at 1 month; (vi) exclusion of ERP recorded before day 7; and (vii) alternative outcome definitions (GOS ≥ 3, or CRS-R total score ≥ 20 at 6 months). Subgroup analyses refitted the multimodal model within strata of etiology, age and FOUR score and compared the AUCs; the interaction between etiology and all predictors was tested with a likelihood-ratio test. Two-sided *p* < 0.05 was considered statistically significant.

## Results

### Baseline characteristics

#### Demographic and clinical data

A comparison of baseline characteristics between the two prognostic groups is presented in Table 1. Significant differences (*p* < 0.05) were observed in age, FOUR score, Fz_MMN_A, Cz_MMN_A, Fz_P300_A, cerebral herniation, etiology, hydrocephalus, and st-SCS surgery. No significant differences were found in Fz_MMN_L, Cz_MMN_L, Fz_P300_L, Cz_P300_L, Cz_P300_A, skull defect, sex, or Rotterdam CT score (all *p* > 0.05).

**Table 1:** Baseline characteristics of the study population.

Variable	Overall (*n* = 227)	eMCS not achieved (*n* = 109)	eMCS achieved (*n* = 118)	P value
Age, years	55.64 (15.55)	58.45 (13.24)	53.05 (17.06)	0.009*
FOUR score	6.77 (3.24)	6.06 (2.96)	7.42 (3.36)	0.001*
Fz_MMN_L, ms	163.24 (48.10)	162.24 (47.42)	164.16 (48.91)	0.764
Fz_MMN_A, μV	−2.03 (1.52)	−1.73 (1.44)	−2.31 (1.54)	0.004*
Cz_MMN_L, ms	165.64 (47.93)	161.03 (47.23)	169.90 (48.38)	0.164
Cz_MMN_A, μV	−1.91 (1.37)	−1.68 (1.24)	−2.12 (1.45)	0.015*
Fz_P300_L, ms	287.50 (53.76)	288.89 (57.29)	286.21 (50.49)	0.709
Fz_P300_A, μV	3.49 (3.05)	2.82 (2.48)	4.12 (3.38)	0.001*
Cz_P300_L, ms	290.35 (62.87)	295.85 (78.17)	285.27 (44.05)	0.206
Cz_P300_A, μV	3.10 (2.74)	2.78 (2.26)	3.39 (3.09)	0.093
Cerebral herniation, *n* (%)				0.035*
No	148 (65.2)	63 (57.8)	85 (72.0)	
Yes	79 (34.8)	46 (42.2)	33 (28.0)	
Hydrocephalus, *n* (%)				0.011*
No	174 (76.7)	75 (68.8)	99 (83.9)	
Yes	53 (23.3)	34 (31.2)	19 (16.1)	
Skull defect, *n* (%)				0.074
No	115 (50.7)	48 (44.0)	67 (56.8)	
Yes	112 (49.3)	61 (56.0)	51 (43.2)	
Etiology, *n* (%)				< 0.001*
nTBI	105 (46.3)	66 (60.6)	39 (33.1)	
TBI	122 (53.7)	43 (39.4)	79 (66.9)	
Sex, *n* (%)				0.770
Female	78 (34.4)	39 (35.8)	39 (33.1)	
Male	149 (65.6)	70 (64.2)	79 (66.9)	
st-SCS surgery, *n* (%)				0.013*
No	206 (90.7)	93 (85.3)	113 (95.8)	
Yes	21 (9.3)	16 (14.7)	5 (4.2)	
Rotterdam CT score, *n* (%)				0.911
1	3 (1.3)	1 (0.9)	2 (1.7)	
2	41 (18.1)	18 (16.5)	23 (19.5)	
3	74 (32.6)	34 (31.2)	40 (33.9)	
4	46 (20.3)	24 (22.0)	22 (18.6)	
5	51 (22.5)	25 (22.9)	26 (22.0)	
6	12 (5.3)	7 (6.4)	5 (4.2)	
Consciousness level at 1 month, *n* (%)				<0.001
Coma	39 (17.2)	31 (28.4)	8 (6.8)	
Vegetative state	80 (35.2)	53 (48.6)	27 (22.9)	
MCS-	62 (27.3)	22 (20.2)	40 (33.9)	
MCS+	18 (7.9)	3 (2.8)	15 (12.7)	
eMCS	28 (12.3)	0 (0.0)	28 (23.7)	
Consciousness level at 6 months, *n* (%)				<0.001
Coma	15 (6.6)	15 (13.8)	0 (0.0)	
Vegetative state	35 (15.4)	35 (32.1)	0 (0.0)	
MCS-	38 (16.7)	38 (34.9)	0 (0.0)	
MCS+	21 (9.3)	21 (19.3)	0 (0.0)	
eMCS	118 (52.0)	0 (0.0)	118 (100.0)	
CRS-R total score, median (IQR)				
At 1 month	7 (3–12)	4 (2–7)	11 (6–19)	<0.001
At 3 months	17 (7–23)	7 (3–11)	23 (21–23)	<0.001
At 6 months	19 (8–23)	8 (4–12)	23 (22–23)	<0.001
Age, years, median (IQR) [range]	59 (49–66) [3–85]	59 (51–68) [3–85]	56 (48–64) [3–80]	0.036
Age < 18 years, *n* (%)	9 (4.0)	1 (0.9)	8 (6.8)	0.037
Time from onset to admission, days, median (IQR)	0 (0–2)	0 (0–2)	0 (0–1)	0.658
Interval from onset to ERP, days, median (IQR)	19 (13–25)	19 (13–25)	20 (12–24)	0.621
MRI performed, *n* (%)	97 (42.7)	39 (35.8)	58 (49.2)	0.057
Length of clinical stay, days, median (IQR)	59 (33–105)	54 (29–106)	62 (41–103)	0.301

Data are presented as mean (SD) for continuous variables and *n* (%) for categorical variables. Wilcoxon rank-sum test was used for continuous variables; Fisher’s exact test or Pearson’s chi-squared test was used for categorical variables. *p < 0.05. Fz_MMN_L, MMN latency at Fz; Fz_MMN_A, MMN amplitude at Fz; Cz_MMN_L, MMN latency at Cz; Cz_MMN_A, MMN amplitude at Cz; Fz_P300_L, P300 latency at Fz; Fz_P300_A, P300 amplitude at Fz; Cz_P300_L, P300 latency at Cz; Cz_P300_A, P300 amplitude at Cz. GOS, Glasgow Outcome Scale, recorded at the 6-month follow-up (1 = death, 2 = vegetative state, 3 = severe disability, 4 = moderate disability, 5 = good recovery); 46 patients (20.3%) were recorded as GOS 1 (death). The consciousness level recorded at 1, 3 and 6 months is the highest level reached by that time, so a patient who died before the 6-month assessment retains the last recorded level.

### Univariate and multivariate logistic regression

#### Study population and timing of the assessment

Of 436 patients with severe brain injury in whom ERP examination was performed, 227 patients met the eligibility criteria and were analyzed (Figure 1); the remaining 209 patients were excluded, most commonly because the ERP examination had been performed outside the 1-month post-onset window (*n* = 183), with smaller numbers excluded because they did not meet the inclusion criteria (*n* = 19; this category includes patients in whom no MMN or P300 could be identified), because they were lost to follow-up or had incomplete 6-month data (*n* = 6), or for another reason (*n* = 1). The clinical variables (age, etiology, FOUR score, cerebral herniation, hydrocephalus, st-SCS surgery and the Rotterdam CT score) were all recorded at admission, a median of 0 days after injury (IQR 0–2, range 0–30), and the Rotterdam CT score was read on that admission scan; the electrophysiological variables were recorded a median of 19 days (IQR 13–25, range 2–31) after injury, and the interval did not differ between the two groups (19 days [IQR 13–25] in patients who did not reach eMCS vs. 20 days [IQR 12–24] in those who did; *p* = 0.621). At the 1-month assessment 39 patients (17.2%) were in coma, 80 (35.2%) in VS, 62 (27.3%) in MCS-, 18 (7.9%) in MCS + and 28 (12.3%) had already reached eMCS (Table 1). At 3 months, 96 of the 118 patients who eventually reached eMCS (81.4%) had already done so, and the remaining 22 patients reached eMCS between 3 and 6 months. At 6 months, 118 patients (52.0%) had reached eMCS and 109 (48.0%) had not (Table 1; Supplementary Table S6). The interval between onset and ERP examination was not associated with the outcome in univariable analysis (Supplementary Table S3).

Univariate logistic regression (with eMCS at 6 months as the dependent variable) identified the following significant predictors: age, FOUR score, Fz_MMN_A, Cz_MMN_A, Fz_P300_A, cerebral herniation, etiology, hydrocephalus, and st-SCS surgery (all *p* < 0.05; Table 2). In a multivariable binary logistic regression containing all nine candidate predictors, age (*p* = 0.007), FOUR score (*p* < 0.001), Fz_P300_A (*p* = 0.012) and etiology (*p* = 0.016) were independently associated with the outcome, whereas cerebral herniation, hydrocephalus, st-SCS surgery, Fz_MMN_A and Cz_MMN_A were not (Table 2; Supplementary Table S1; Figure 2). To evaluate the reliability of the multivariate findings, we tested for potential interaction effects between Fz_MMN_A and key clinical predictors (FOUR score and age). The interaction terms were not statistically significant (FOUR x Fz_MMN_A: *p* = 0.924; Age x Fz_MMN_A: *p* = 0.157), indicating that the prognostic effect of Fz_MMN_A is consistent across different levels of consciousness severity and age groups (Table 3).

**Table 2:** Univariate and multivariate logistic regression analysis.

Variable	Univariate OR (95% CI)	Univariate P	Multivariate OR (95% CI)	Multivariate P
Age	0.976 (0.959–0.994)	0.011*	0.969 (0.947–0.992)	0.007*
FOUR score	1.152 (1.052–1.261)	0.002*	1.190 (1.076–1.316)	< 0.001*
Fz_MMN_L	1.001 (0.995–1.006)	0.763	—	—
Fz_MMN_A	0.766 (0.635–0.923)	0.005*	0.812 (0.584–1.129)	0.216
Cz_MMN_L	1.004 (0.998–1.009)	0.164	—	—
Cz_MMN_A	0.782 (0.640–0.957)	0.017*	1.032 (0.734–1.450)	0.856
Fz_P300_L	0.999 (0.994–1.004)	0.707	—	—
Fz_P300_A	1.170 (1.059–1.292)	0.002*	1.157 (1.033–1.295)	0.012*
Cz_P300_L	0.997 (0.993–1.002)	0.217	—	—
Cz_P300_A	1.089 (0.985–1.204)	0.097	—	—
Herniation (Y vs. N)	0.532 (0.306–0.925)	0.025*	0.614 (0.319–1.180)	0.143
Hydrocephalus (Y/N)	0.423 (0.224–0.800)	0.008*	0.639 (0.300–1.359)	0.244
Skull defect (Y/N)	0.599 (0.354–1.013)	0.056	—	—
Etiology (TBI/nTBI)	3.109 (1.807–5.350)	< 0.001*	2.142 (1.154–3.976)	0.016*
Sex (M vs. F)	1.129 (0.652–1.953)	0.665	—	—
st-SCS (Y vs. N)	0.257 (0.091–0.728)	0.011*	0.424 (0.138–1.304)	0.134
Rotterdam CT score	0.893 (0.719–1.110)	0.309	—	—

The multivariable column is the full nine-variable model, in which all nine candidate predictors were entered simultaneously; no data-driven variable selection was applied. The symbol * means *p* < 0.05 (statistically significant).

**Figure 2:**
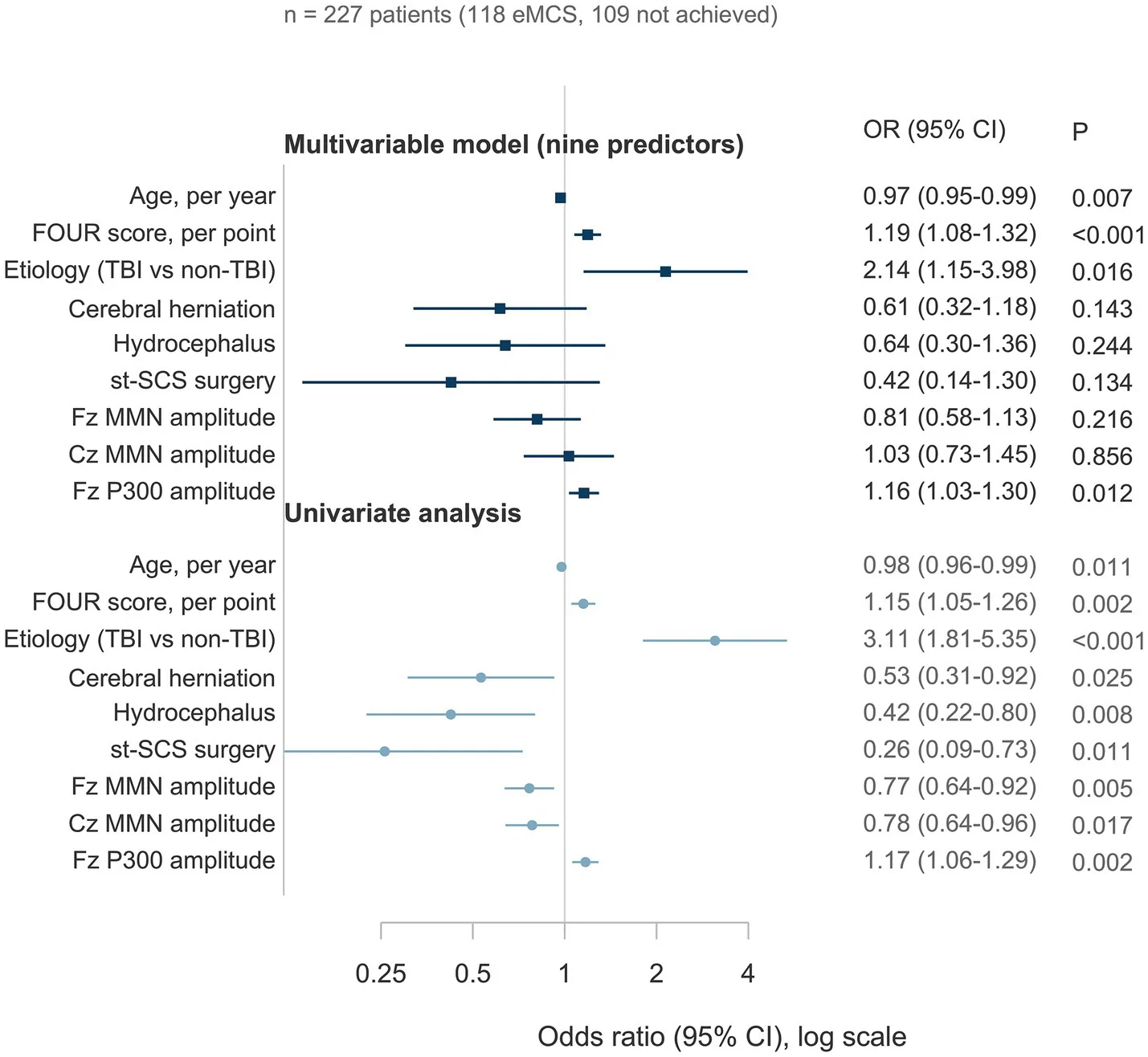
Forest plot of the univariate and multivariable logistic regression analyses of the nine candidate predictors (227 patients; 118 who achieved and 109 who did not achieve emergence from the minimally conscious state at 6 months). Odds ratios with 95% confidence intervals are plotted on a logarithmic scale and the Wald *p* value of each predictor is given on the right. The multivariable model contained all nine candidate predictors.

**Table 3:** Interaction analysis between Fz_MMN_A and clinical predictors.

Interaction term	Beta	Standard error	Wald Z	*p*-value
FOUR x Fz_MMN_A	−0.00146	0.01527	−0.096	0.924
Age x Fz_MMN_A	−0.00780	0.00551	−1.417	0.157

### Prediction model development and evaluation

Three models were used to predict whether patients would reach eMCS at 6 months (Supplementary Table S1; Figure 3). The clinical model contained the six acute-phase clinical variables (age, etiology, FOUR score, cerebral herniation, hydrocephalus and st-SCS surgery) and had an AUC of 0.745 (95% CI 0.681–0.809). The electrophysiological model contained the three ERP amplitude measures (Fz_MMN_A, Cz_MMN_A and Fz_P300_A) and had an AUC of 0.654 (95% CI 0.583–0.726). The multimodal model contained all nine variables and had an AUC of 0.772 (95% CI 0.711–0.832). The multimodal model was therefore the best performing model, and its AUC was slightly higher than that of the clinical model (ΔAUC = 0.027), although this difference was not statistically significant (DeLong test *p* = 0.115); the difference between the multimodal and the electrophysiological model was larger and was statistically significant (DeLong test *p* = 0.001; Figure 3). The variables of the three models are listed explicitly in Supplementary Table S1.

**Figure 3:**
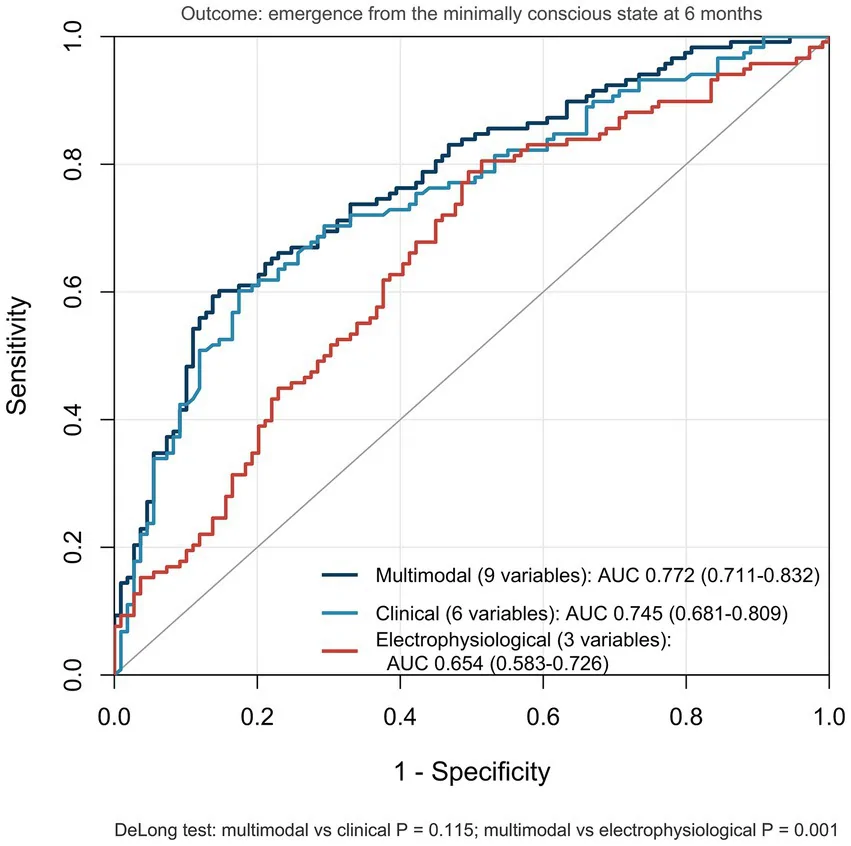
Receiver operating characteristic curves of the clinical (six variables), electrophysiological (three variables), and multimodal (nine variables) models for emergence from the minimally conscious state at 6 months (227 patients). The area under the curve with its 95% confidence interval is given for each model and the DeLong test comparisons are shown in the panel.

### Internal validation and calibration

The three models were internally validated (Supplementary Table S2). After bootstrap correction (1,000 resamples) the AUC was 0.735 for the multimodal model, 0.720 for the clinical model and 0.638 for the electrophysiological model, corresponding to optimism-corrected estimates of 0.735 and 0.720, respectively, (optimism 0.037 and 0.025). Repeated 10-fold cross-validation gave similar values (0.730, 0.711 and 0.621). Calibration was adequate: the Hosmer-Lemeshow test was not significant for any model (multimodal chi-square = 6.12, df = 8, *p* = 0.634; clinical chi-square = 5.45, df = 8, *p* = 0.708; electrophysiological chi-square = 10.11, df = 8, *p* = 0.257), the Brier score was 0.194 for the multimodal model (scaled Brier score, an R2 analogue, 0.223), and the bootstrap-corrected calibration slope was 0.80, indicating slight overfitting of the predicted probabilities. Calibration curves for the three models are shown in Figure 4.

**Figure 4:**
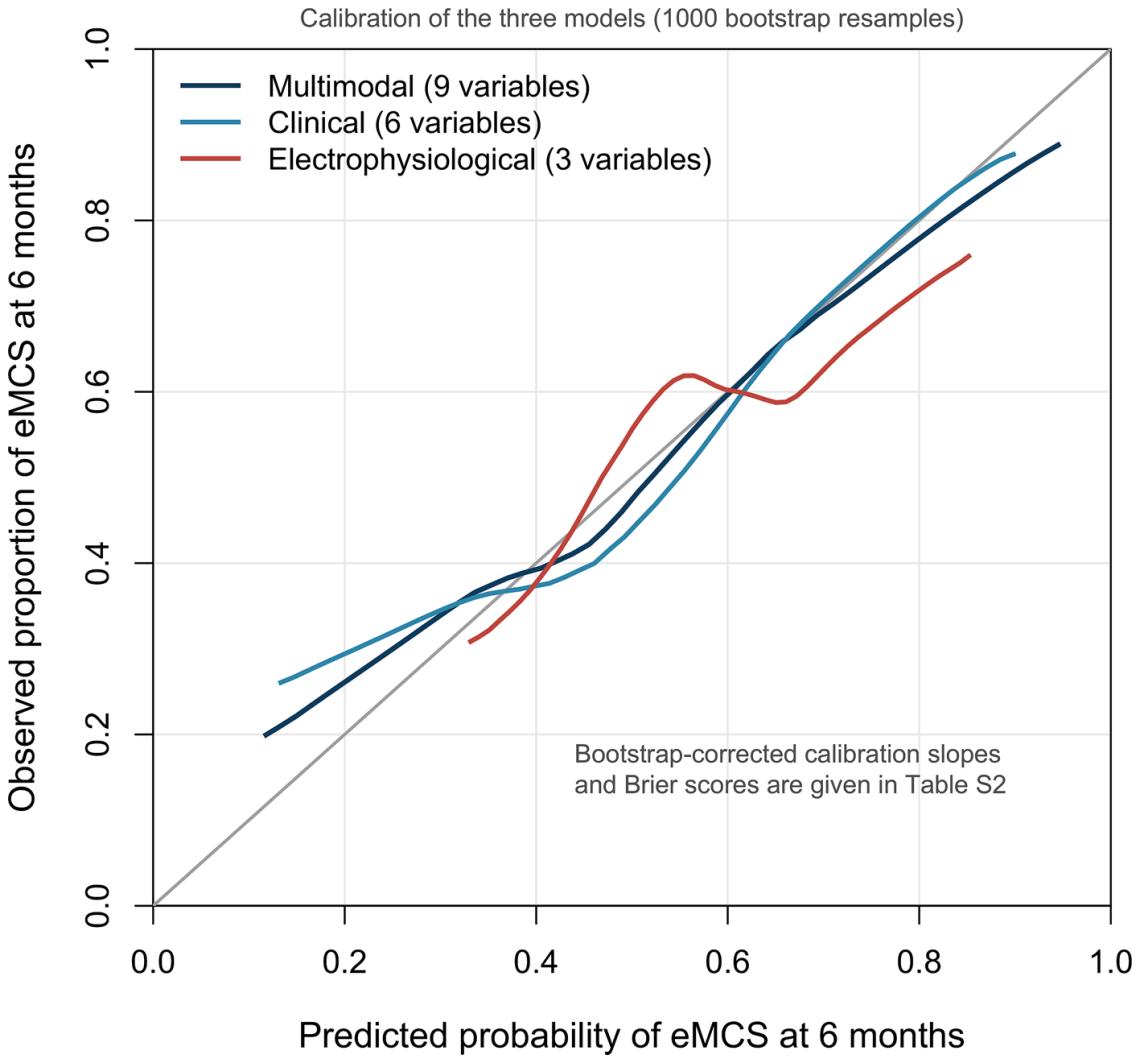
Bootstrap-corrected calibration curves of the clinical, electrophysiological and multimodal models (1,000 bootstrap resamples). The gray line indicates perfect calibration.

The calibration curve of the multimodal model (Figure 4) showed good agreement between predicted and observed probabilities across the majority of the risk spectrum. In the low-to-moderate risk range (0–0.25), the model slightly overestimated risk; in the moderate-to-high risk range (0.25–0.75), predicted probabilities closely aligned with observed outcomes; at very high risk (> 0.75), a slight underestimation was observed. Overall, the bias-corrected calibration curve remained close to the ideal 45-degree line, supporting the reliability of the multimodal model for clinical application.

Decision curve analysis (Figure 5) compared the net benefit of the clinical, electrophysiological and multimodal models with the two reference strategies ‘treat all’ and ‘treat none’. The multimodal model had a higher net benefit than both reference strategies and than the clinical model across threshold probabilities of approximately 5 to 50%, and it crossed the ‘treat all’ line at a threshold probability of approximately 20.1%. Above a threshold of about 50% the model no longer outperformed ‘treat all’, so the range of clinical usefulness is restricted. Decision curve analysis describes the statistical performance of a prediction rule across hypothetical threshold probabilities; net benefit is not a measure of treatment effect and the analysis implies no recommendation about treatment. It does not indicate that the model identifies patients who ‘require’ treatment, and the predicted probability must not be used as the basis for decisions about the withdrawal or withholding of treatment, which should continue to be made by the treating team on clinical grounds, in discussion with families and according to local law and guidelines. The intended use of the model is to support structured, repeated, multidisciplinary prognostic counseling of families and to inform the planning of active care, not to justify its limitation.

**Figure 5:**
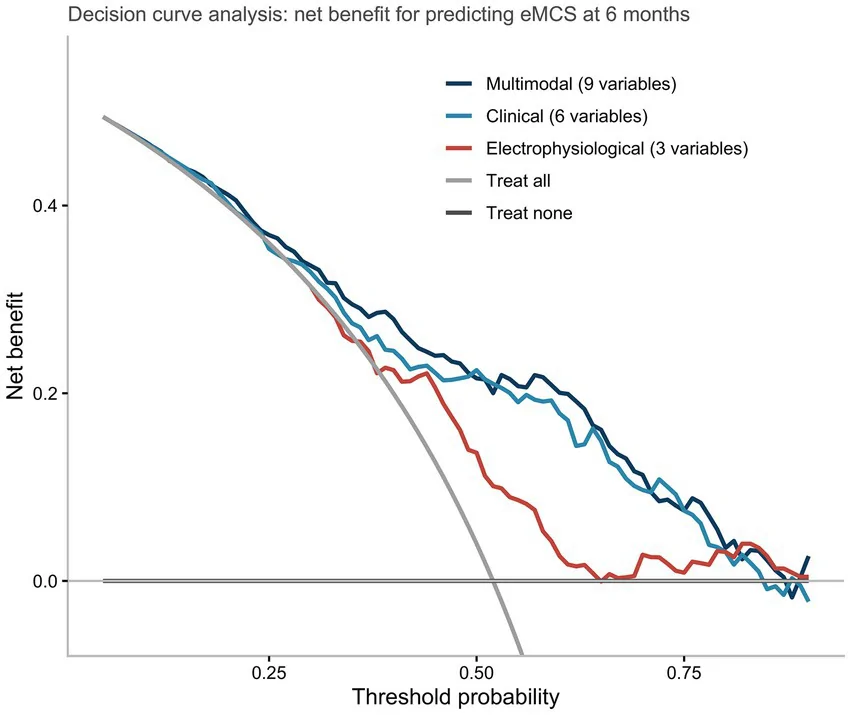
Decision curve analysis of the three prediction models, compared with the strategies of treating all patients and treating none, across threshold probabilities.

The multimodal model was visualized as a nomogram (Figure 6), incorporating all 9 acute-phase predictors. Each predictor is assigned a score, and the total score corresponds to the predicted probability of consciousness improvement at 6 months. Variable importance ranking (Figure 7) identified the FOUR score, age, Fz_MMN_A, and etiology as the most influential contributors to the model.

**Figure 6:**
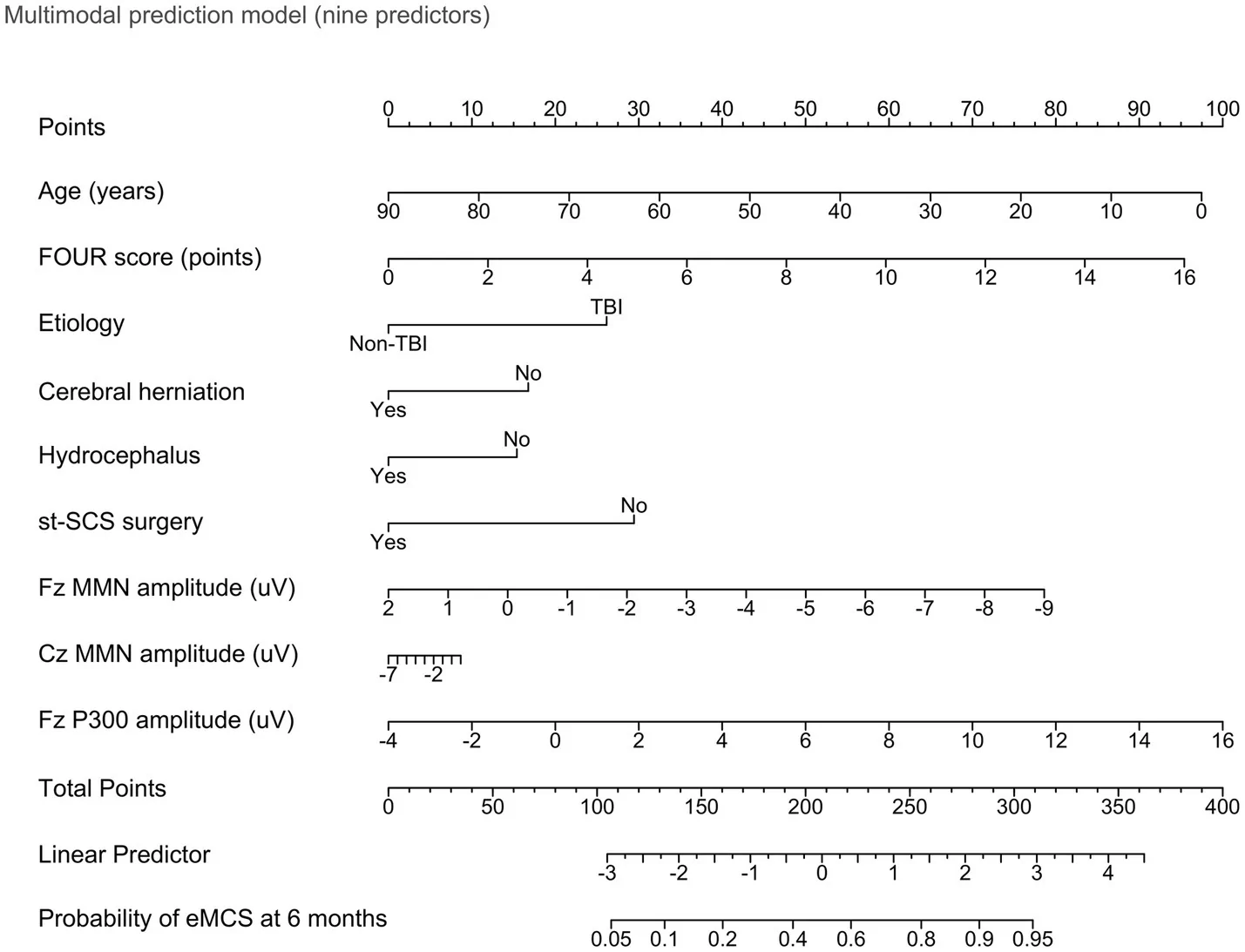
Nomogram of the multimodal prediction model, which converts the value of each of the nine predictors into a predicted probability of emergence from the minimally conscious state at 6 months.

**Figure 7:**
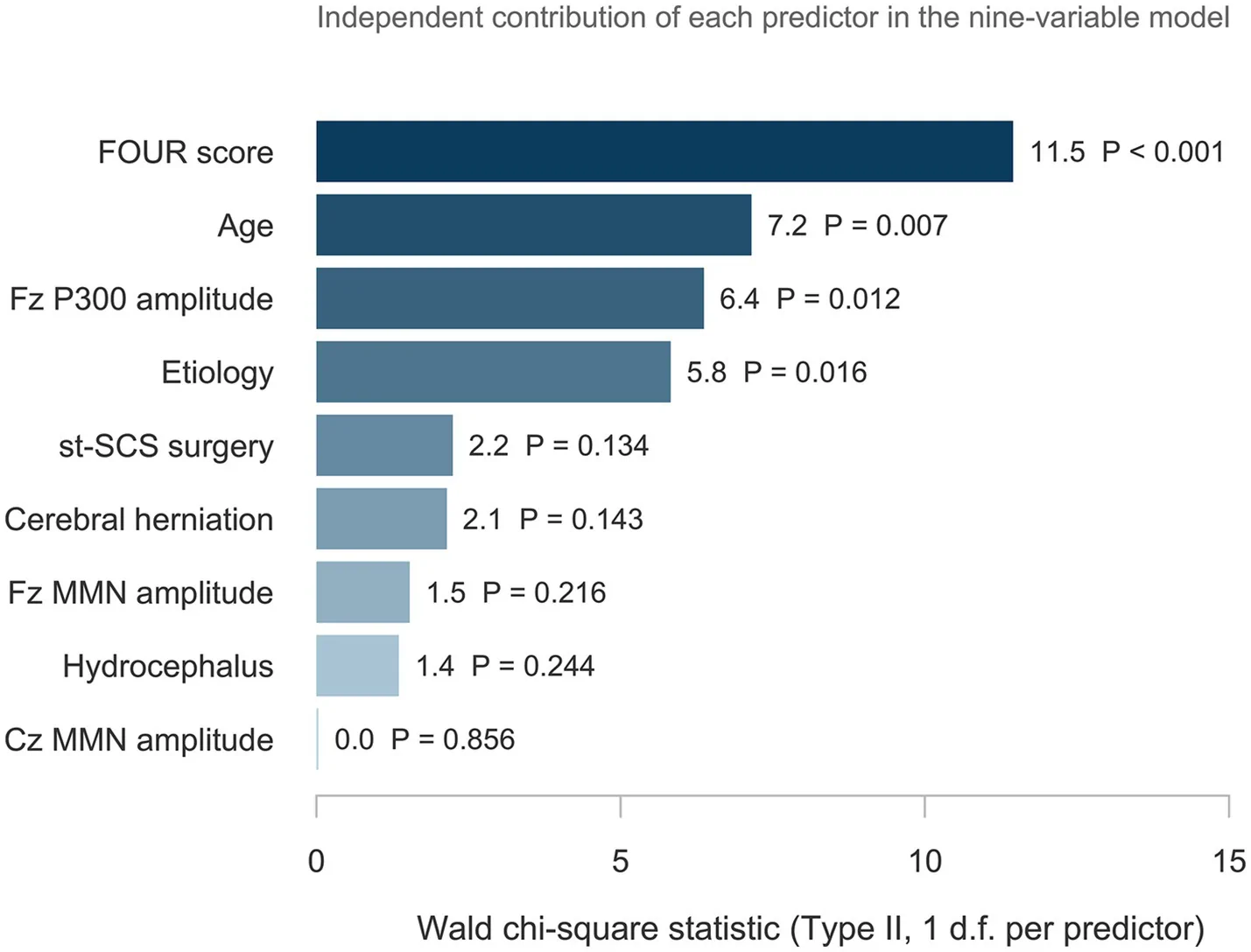
Variable importance in the nine-variable model, ranked by the Wald chi-square statistic; the statistic and the corresponding *p* value are given for each predictor.

A risk reclassification scatter plot (Figure 8) visualized the change in predicted probability when adding electrophysiological indicators to the clinical model. Each dot represents an individual patient, with the x-axis showing the clinical model’s predicted probability of favorable prognosis and the y-axis showing the multimodal model’s predicted probability. The continuous net reclassification improvement (NRI) was 0.317 (*p* = 0.015), and the integrated discrimination improvement (IDI) was 0.039 (*p* = 0.003), indicating that the multimodal model significantly improved risk stratification compared to the clinical model alone.

**Figure 8:**
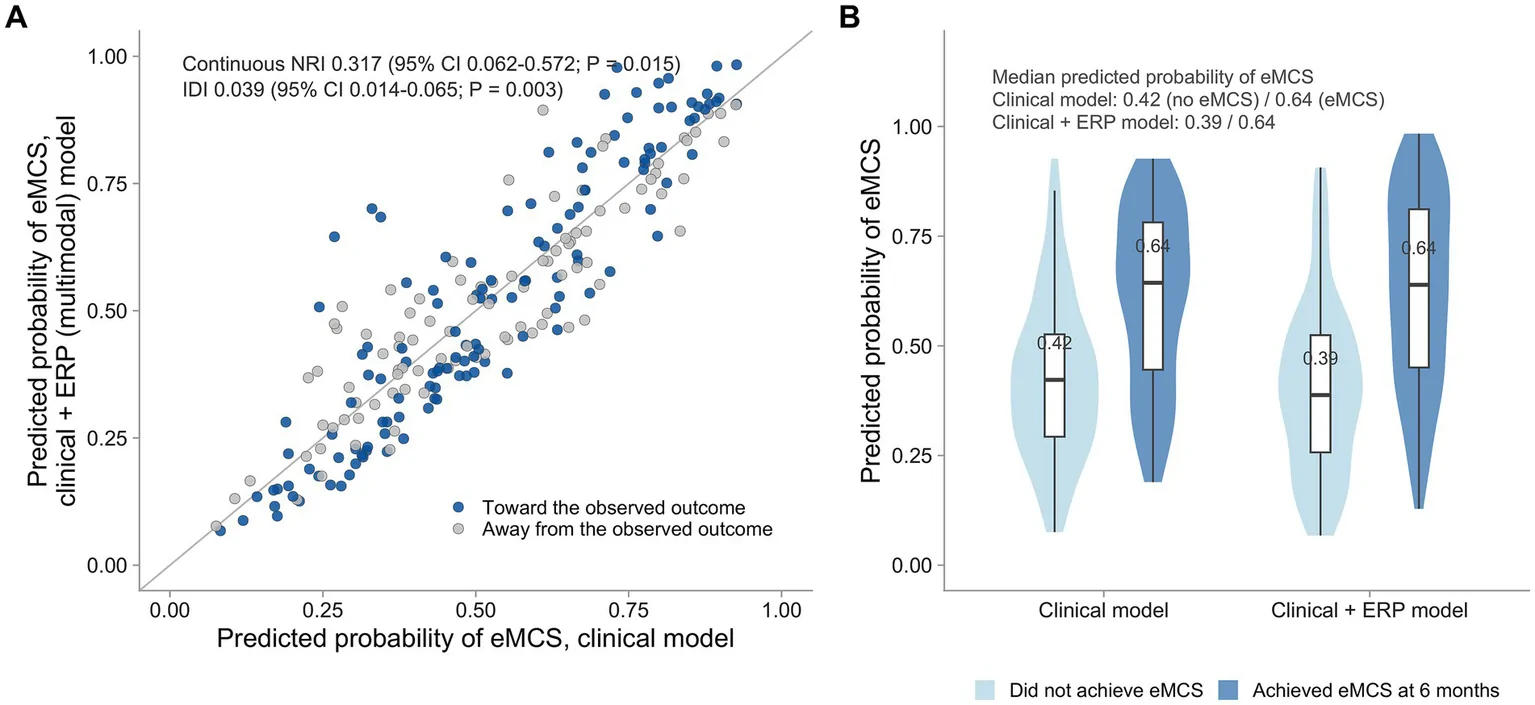
**(A)** Risk reclassification scatter plot showing the individual predicted probabilities of the clinical and the multimodal model for each patient (*n* = 227); the vertical distance from the diagonal indicates the change in predicted probability, with patients whose predicted probability moved toward the observed outcome shown in blue and those who moved away shown in gray; the annotations give the continuous NRI and the IDI with their 95% confidence intervals. **(B)** Distributions of the predicted probability of eMCS in the clinical model and the clinical + ERP (multimodal) model, shown as violin plots with box plots for patients who did not achieve eMCS and patients who achieved eMCS at 6 months; the numbers above the violins are the median predicted probabilities in each group.

### Sensitivity analyses

The results of the multimodal model were stable in all pre-specified sensitivity analyses (Supplementary Table S3). Adjustment for the interval between injury and ERP recording did not change discrimination (AUC = 0.771; odds ratio per day 1.014, *p* = 0.541), and the interval alone was not discriminative (AUC = 0.481). Restriction to ERP performed within 28 days of onset gave an AUC of 0.775, and analysis by timing stratum gave 0.892 (*n* = 71, median 10 days) for ERP recorded 2–14 days after onset and 0.750 (*n* = 156, median 23 days) for ERP recorded 15–31 days after onset, so the predictive information is present early and does not depend on late recordings. Exclusion of patients younger than 18 years (AUC = 0.762), exclusion of patients who died before the 6-month assessment (AUC = 0.787), exclusion of ERP recorded before day 7 (AUC = 0.767) and the complete-case analysis (*n* = 220; AUC = 0.771, 95% CI 0.709–0.833) gave results similar to the primary analysis. Because patients who reached eMCS by the 1-month assessment had, by definition, already reached the outcome, a sensitivity analysis excluded these 28 patients and was restricted to patients who were in VS or MCS at 1 month (*n* = 199 with 90 events; AUC = 0.722, 95% CI 0.652–0.793). Alternative outcome definitions gave similar discrimination (GOS ≥ 3 at 6 months, AUC = 0.765, 95% CI 0.704–0.826; CRS-R total ≥ 20 at 6 months, AUC = 0.760, 95% CI 0.698–0.823; Supplementary Table S3), indicating that the results do not depend on the specific definition of the primary outcome. Analyses in which the variable-selection procedures were applied to the same data set gave similar discrimination (backward elimination at *p* < 0.05, AUC = 0.753, 95% CI 0.691–0.816; backward elimination by AIC, AUC = 0.768, 95% CI 0.707–0.829; neither significantly different from the pre-specified nine-variable model, DeLong test *p* = 0.196 and *p* = 0.564; Supplementary Table S3).

### Subgroup analysis

The model was refitted within each stratum to avoid attenuation bias (Figure 9). Discrimination was similar in patients with TBI (*n* = 122, 79 events; AUC = 0.794, 95% CI 0.714–0.873) and in patients with non-traumatic injury (*n* = 105, 39 events; AUC = 0.733, 95% CI 0.635–0.831), and the difference between the two ROC curves was not statistically significant (*p* = 0.346). A likelihood-ratio test comparing a model containing etiology by predictor interaction terms with the main-effects model was also not significant (*p* = 0.271). AUCs were comparable across age and FOUR score strata (Supplementary Table S4). These analyses were performed in a single cohort of 227 patients with 39 to 79 events per stratum, and they are therefore underpowered to exclude moderate differences in performance between strata; the results should be read as an absence of strong evidence of heterogeneity rather than as evidence of equivalence.

**Figure 9:**
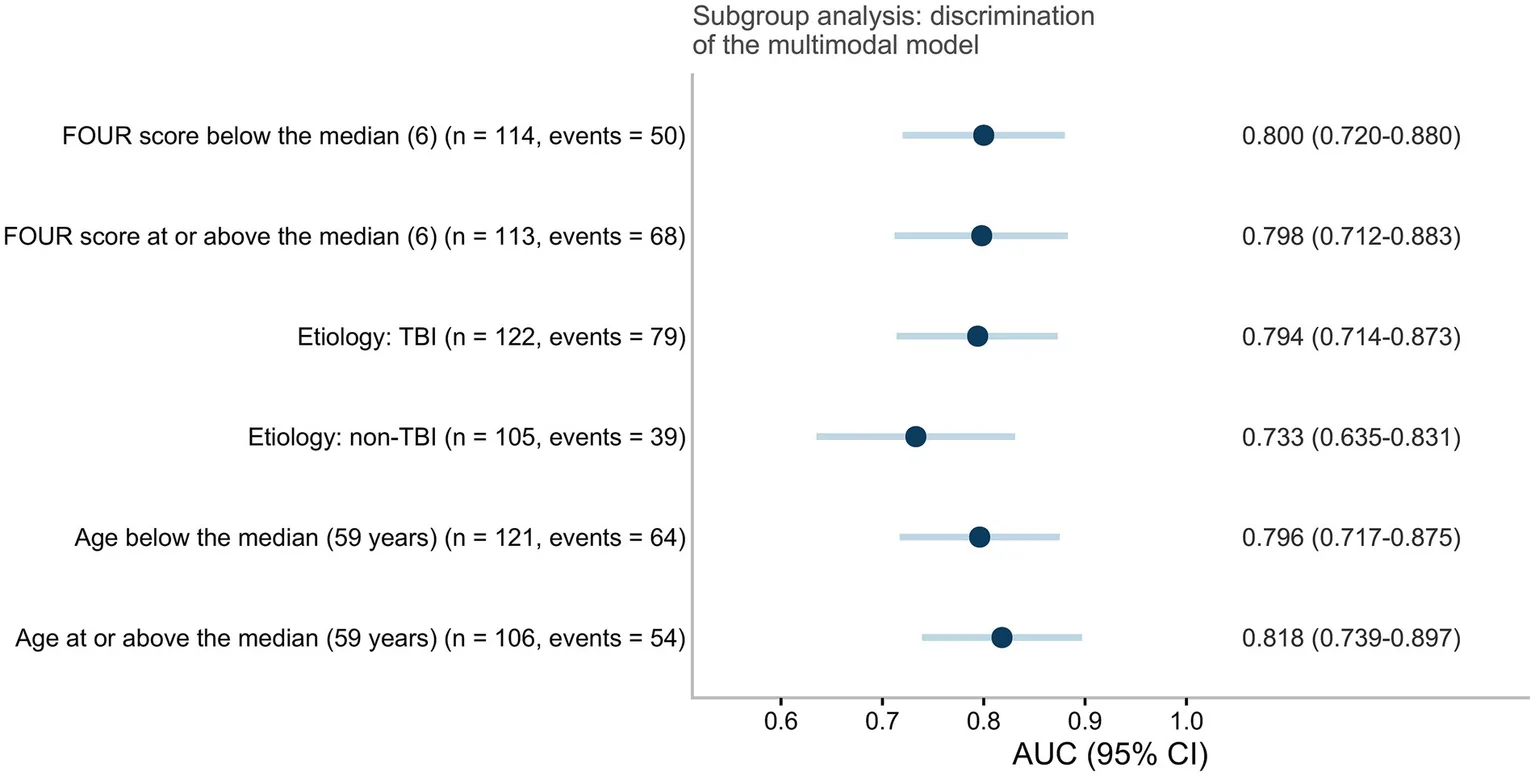
Discrimination of the multimodal model within subgroups defined by etiology, age, and FOUR score. The model was refitted within each stratum. The area under the curve with its 95% confidence interval is shown together with the number of patients and the number of events in that stratum. Age and FOUR score were dichotomized at their medians.

### Consciousness outcome and functional status at 6 months

At 6 months the two groups differed markedly in the CRS-R total score (median 8 [IQR 4–12] in patients who did not reach eMCS vs. 23 [IQR 22–23] in those who did; *p* < 0.001) and in the distribution of CRS-R total scores (Figure 10). The definition of eMCS is based on functional communication or functional object use rather than on the total score, and 37 of the 118 patients who reached eMCS (31.4%) had a total score below 23. Functional status was less favorable than consciousness status: among the 118 patients who reached eMCS, the GOS at 6 months was 3 in 57 patients (48.3%), 4 in 19 (16.1%), and 5 in 40 (33.9%), whereas 2 patients (1.7%) had a GOS of 1 or 2; both of these patients had already met the criteria for eMCS at the 3-month assessment, and their status at 6 months was recorded as a vegetative state in one and as death in the other. Among the 109 patients who did not reach eMCS, the GOS was 1 in 45 (41.3%), 2 in 55 (50.5%) and 3 in 9 (8.3%); no patient in this group had a GOS of 4 or 5 (*p* < 0.001 for the comparison of the GOS distribution between groups; Supplementary Table S6). In total, 46 patients (20.3%) were recorded as GOS 1 (death) at the 6-month follow-up. Recovery of consciousness therefore did not equate to functional independence in this cohort.

**Figure 10:**
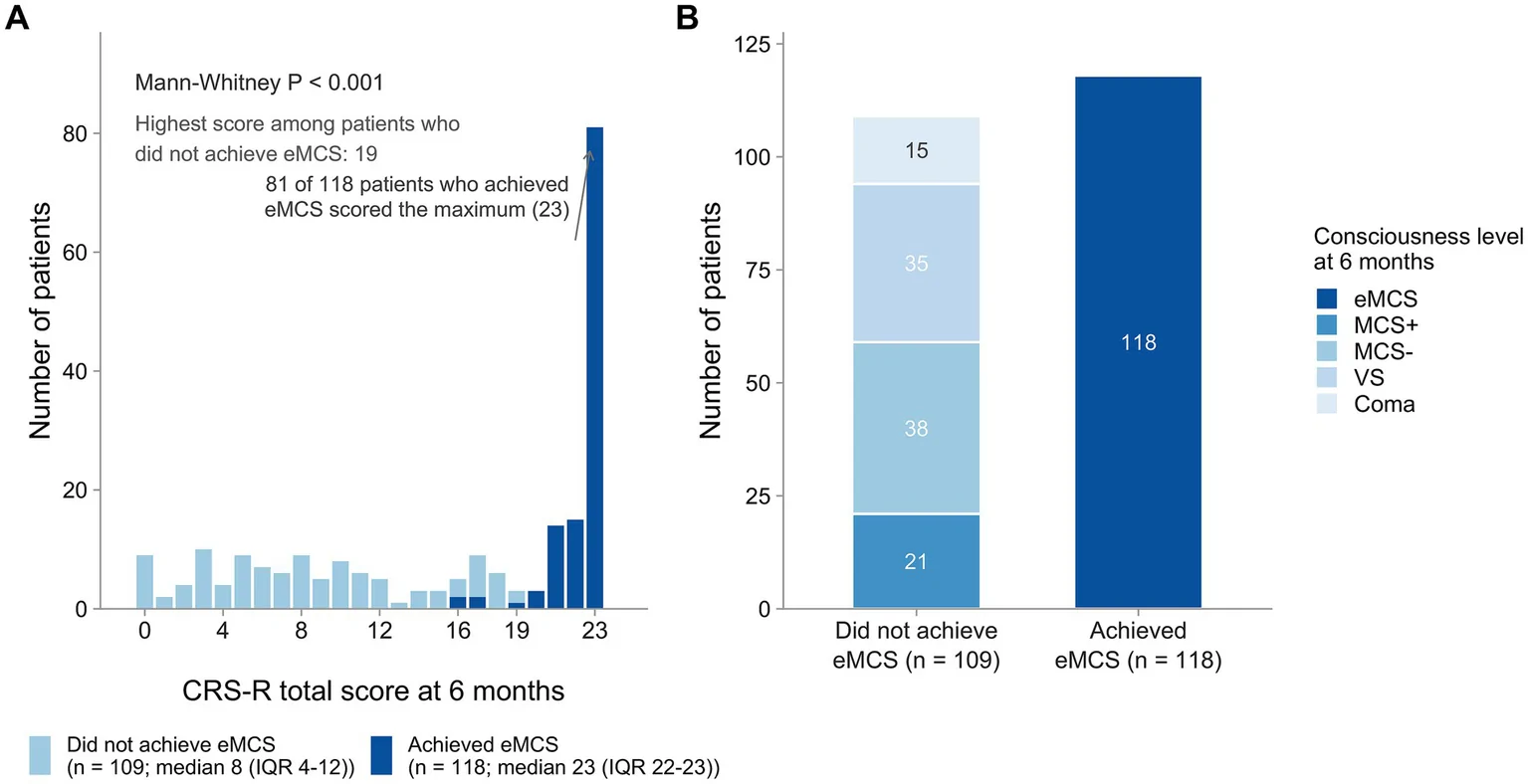
Coma Recovery Scale-Revised (CRS-R) total score at 6 months **(A)** and the consciousness level of each group **(B)**. **(A)** The bars give the number of patients at each CRS-R total score, separately for patients who did and did not reach eMCS; the median score of each group is given beneath the group label and the Mann–Whitney *p* value is shown in the panel. **(B)** The segments give the number of patients in a coma, in a vegetative state (VS), in a minimally conscious state minus (MCS-) or plus (MCS+), and in emergence from MCS (eMCS) within each group. In **(B)** the bars show the highest consciousness level recorded in the registry by each time point rather than a physiological state present at the 6-month visit, and the lowest category is the registry label recorded as coma, which should not be read as physiological coma at 6 months; these patients had a CRS-R total score of 0–3 at every assessment and, at the 6-month time point, 10 were recorded as GOS 1 (death) and 5 as GOS 2 (vegetative state) (see Limitations).

## Discussion

With advances in medical technology, more patients with severe brain injuries survive the acute phase. Clinicians and families then face a critical question: will the patient regain consciousness (14)? Song et al. systematically reviewed 21 prognostic models involving 1,201 DoC patients. They concluded that each individual technique has inherent strengths and limitations, and that multimodal integration has the potential to improve prediction accuracy (15, 16).

DoC patients present unique challenges. Transporting them for prolonged examinations carries substantial risk. Intracranial implants such as aneurysm clips, fixation materials, cranioplasty materials, and shunt tubes can affect MRI signal quality (17, 18). PET imaging is prohibitively expensive and unavailable in most hospitals (19). Therefore, bedside examinations that are simple and easy to implement are preferable for broader clinical adoption. These include somatosensory evoked potentials (SEP), ERP, electroencephalography (EEG), and brainstem auditory evoked potentials (BAEP) (20).

Gui et al. used bedside EEG to investigate neural representations of language processing in DoC patients. By combining these representations with machine learning methods, they achieved consciousness state diagnosis and recovery prediction. While these findings provide new clinical reference points, their widespread implementation requires further refinement (21).

Most previous studies have used chronic-phase examination results for consciousness assessment and long-term prognosis prediction in DoC patients (8, 20–25). Relatively few have leveraged acute-phase data. To our knowledge, this study is among the first to construct a multimodal prediction model using acute-phase indicators (age, etiology, FOUR score, and acute-phase ERP measures) to predict whether patients will reach eMCS at 6 months post-onset. This approach enables early prognostication of long-term outcomes in DoC patients, which has direct implications for clinical decision-making and family counseling.

Analysis of baseline data between the two prognostic groups revealed significant differences in age, FOUR score, Fz_MMN_A, Cz_MMN_A, Fz_P300_A, cerebral herniation, etiology, hydrocephalus, and st-SCS surgery (all *p* < 0.05; Table 1). Further regression analyses confirmed that etiology, age, and acute-phase FOUR score were independently associated with prognosis. Among ERP components, Fz_P300_A emerged as an independent predictor in multivariate analysis. Fz_MMN_A and Cz_MMN_A, though significant in univariate analysis, did not retain independence. This may partly reflect the correlation between the two MMN amplitudes (*r* = 0.70; variance inflation factor 2.4 in the multimodal model) and the fact that these measures arise from overlapping processes along the auditory processing hierarchy; however, the degree of collinearity was moderate, and the more likely explanation is that the MMN amplitudes carry less independent prognostic information than the P300 amplitude in this cohort. Replication in an independent cohort is required before this difference between ERP components is interpreted as a neurophysiological finding.

We constructed three prediction models and compared their performance. The multimodal model, which incorporated all nine candidate predictors, achieved the highest discrimination (AUC = 0.772), slightly exceeding the clinical model (AUC = 0.745) and clearly exceeding the electrophysiological model (AUC = 0.654). The ΔAUC of 0.027 did not reach statistical significance (DeLong test *p* = 0.115), which may reflect limited statistical power with 227 patients. Discrimination was maintained after bootstrap correction (multimodal model 0.735; clinical model 0.720) and in repeated 10-fold cross-validation (0.730 and 0.711), indicating that the apparent performance is not simply the result of overfitting in a small sample. Calibration was adequate in both models. In decision curve analysis the multimodal model had a higher net benefit than the reference strategies across threshold probabilities of about 5 to 50% (Figure 5), and reclassification analysis indicated that adding electrophysiological measures changed predicted risks in a direction consistent with better discrimination. These analyses support a modest incremental contribution of ERP measures to clinical prediction rather than a large improvement in accuracy.

The risk reclassification analysis provided further evidence of incremental value (Figure 8). With the clinical model as the reference, the continuous NRI was 0.317 (95% CI 0.062–0.572, *p* = 0.015) and the IDI was 0.039 (95% CI 0.014–0.065, *p* = 0.003; Supplementary Table S5). Because the continuous NRI is a sum of directional changes in predicted probability, it can be inflated by miscalibration, and its components should therefore be examined separately: among the 118 patients who reached eMCS, 62 were assigned a higher probability and 56 a lower probability by the multimodal model (events component 0.051), whereas among the 109 patients who did not reach eMCS, 69 were assigned a lower and 40 a higher probability (non-events component 0.266). The improvement was thus driven mainly by downward reclassification of patients who did not achieve eMCS. The categorical NRI with risk strata of 0.2 and 0.5 was smaller and not statistically significant (0.056, 95% CI -0.055 to 0.167, *p* = 0.325), indicating that the incremental value was not concentrated at particular risk cut-offs. These results are consistent in direction with the systematic review by Song et al. (15, 16), although the incremental improvement was modest and its clinical importance remains to be demonstrated prospectively.

This reclassification has potential practical implications. Among patients assigned an intermediate risk by the clinical model, adding the Fz P300 amplitude changed the estimated probability for the individual patient, which may support the planning of rehabilitation intensity and the content of discussions with families. The model was developed retrospectively in a single center and has not been evaluated prospectively; it therefore provides prognostic information to complement, and not to replace, clinical judgment and the repeated multidisciplinary assessment of the patient, and it should not be used to justify the withdrawal or withholding of treatment.

Subgroup analysis did not show a significant difference in the performance of the multimodal model between patients with TBI and patients with non-traumatic injury (*p* = 0.346; interaction *p* = 0.271). With 39 events in the non-traumatic subgroup, however, the analysis has limited power, and the confidence intervals of the stratum-specific AUCs overlap substantially. These results therefore do not establish that the model performs equally well in all etiological groups; they indicate only that no large difference was detected in this cohort, and performance in individual etiological subgroups requires confirmation in larger, ideally multicenter, cohorts.

Previous studies have established the prognostic value of chronic-phase ERP components (MMN and P300) in DoC. These have been incorporated into the European Academy of Neurology guideline on disorders of consciousness (2020) (3). In our study, Fz_MMN_A, Cz_MMN_A, and Fz_P300_A were all significant in univariate analysis, but only Fz_P300_A remained significant in multivariate analysis. This finding suggests that P300 may carry greater prognostic weight than MMN in the acute phase. P300 reflects longer-latency cognitive processing and the integrity of higher-order neural circuits, whereas MMN primarily indexes pre-attentive sensory processing. Our study extends the literature by applying ERP assessment in the acute phase and combining it with clinical features for multimodal evaluation. This substantially advances the timeline of prognostic assessment compared to prior work focused on chronic-phase data.

We chose the FOUR score over the commonly used Glasgow Coma Scale (GCS) for a specific reason. Many DoC patients require endotracheal intubation and mechanical ventilation. In such patients, the verbal component of the GCS must be replaced with “T” (tubus), which is conventionally converted to a score of 1. However, not all intubated patients have truly lost verbal function. The FOUR score circumvents this limitation and is recommended by European and American guidelines (2, 3).

Consistent with prior work (15, 16), our results confirm that age and etiology play important roles in long-term prognosis. Older age and non-traumatic etiology were associated with worse outcomes.

St-SCS has been widely validated as a therapeutic modality for DoC (12, 13, 26, 27), although its short-term form is relatively new (12, 27). In our study, st-SCS was a significant predictor in univariate but not multivariate analysis. Our team has previously demonstrated positive effects of st-SCS in DoC patients (13). The lack of independent significance in the present analysis may reflect the absence of propensity score matching, since patients who received st-SCS were not matched with comparable controls; in addition, st-SCS was not allocated randomly, so this variable may act as a marker of the severity of the injury, of family preference, or of the treating team’s expectation, and its coefficient should not be interpreted as an estimate of treatment effect. The prognostic value of st-SCS requires evaluation in a randomized or propensity-matched design.

The Rotterdam CT score did not show a significant association with prognosis in either univariate or multivariate analysis. One possible explanation is that all enrolled patients were critically ill with relatively high CT scores, limiting variability. Another is that the Rotterdam CT score was originally designed for TBI patients (28). Its application to nTBI patients in this study may have been inappropriate.

### Consciousness and function as separate outcomes

The distinction between recovery of consciousness and recovery of function is important for the interpretation of our results. In this cohort, 118 patients (52.0%) had reached eMCS by 6 months, but only 40 of them (33.9%) had a GOS of 5 and a further 19 (16.1%) had a GOS of 4, so that 57 patients (48.3%) who were able to communicate or use objects functionally remained severely dependent (GOS 3). Conversely, 9 patients who did not meet the criteria for eMCS had a GOS of 3, reflecting the fact that the GOS integrates motor and cognitive disability as well as consciousness. The agreement between reaching eMCS at 6 months and a GOS of 3 or better was 95.2% (κ = 0.903). Two patients who met the criteria for eMCS were nevertheless recorded with a GOS of 1 or 2: in one the recorded consciousness level was eMCS with a GOS of 2, and in the other, who died, the recorded consciousness level was eMCS with a GOS of 1. A model that predicts emergence from the MCS should therefore not be reported as a model that predicts independence, and conversations with families should distinguish these two outcomes explicitly. This is also why we chose the CRS-R based definition of eMCS, which follows current diagnostic criteria and is not equivalent to a total-score threshold: one third of the patients diagnosed with eMCS had a CRS-R total score below 23. When families ask whether the patient will wake up, the answer that this model can support concerns the recovery of consciousness; the questions of function, dependency and burden of care require separate discussion and separate prognostic information. The burden carried by families of patients with prolonged DoC is substantial and includes unmet needs for information and support (29).

### Limitations and future directions

This study has several limitations. First, it is a single-center retrospective study of 227 patients, and the models were internally validated only, so external validation in an independent, ideally multicenter cohort is required before clinical use and performance in a new setting is likely to be optimistic. The cohort is also a selected one, because an MMN or a P300 had to be identifiable in the ERP recording for inclusion, so patients without a detectable cortical auditory response are not represented, and residual confounding by unmeasured factors cannot be excluded. Second, the outcome was established behaviorally and is therefore subject to misclassification. The CRS-R was administered three times at each time point, with the best of the three scores used, because a single examination misclassifies a substantial proportion of patients with disorders of consciousness (5), and about 40% of patients diagnosed as being in a vegetative state have shown signs of consciousness on the CRS-R (30). Patients who could not attend hospital were assessed by structured telephone follow-up, which is less reliable than direct examination, and missing ER*p* values were imputed; a complete-case analysis gave an almost identical AUC, but imputation rests on assumptions that cannot be fully verified. The outcome is the consciousness level at 6 months, and 28 of the 118 patients who reached eMCS (23.7%) had already done so at 1 month, so that in these patients the acute-phase predictors were recorded after the outcome had effectively occurred; excluding them gives an AUC of 0.722. A 6-month horizon also measures an intermediate rather than a final state, because recovery can continue for 12 months or longer, and this will tend to attenuate rather than to create the associations reported here. Third, the candidate predictors were limited. Imaging was represented only by the Rotterdam CT score, because MRI was available in part of the cohort (97 patients, 42.7%), sequences beyond conventional structural imaging in fewer still, and functional MRI was not acquired with a uniform protocol, so that markers such as diffuse axonal injury, midline shift and cerebral edema were not represented in the models; the Rotterdam CT score itself was not associated with the outcome. The ERP protocol was applied in a clinical rather than a research setting, and the interval between injury and recording varied between 2 and 31 days, although sensitivity analyses restricted to narrower intervals gave similar results. st-SCS was neither randomly allocated nor propensity-matched, so this variable may act as a marker of injury severity or of family preference rather than as an estimate of treatment effect. Fourth, the cohort was heterogeneous. Etiology was analyzed as TBI versus non-TBI, and the non-traumatic group combined ischemic and hemorrhagic stroke (*n* = 94) with hypoxic–ischemic encephalopathy (*n* = 11), in which a cardiac arrest was documented in 9; because these mechanisms differ in the pattern and severity of the injury they produce, pooling them may dilute or distort their prognostic signal, and mechanism-specific models could not be fitted in a group of this size. Nine patients were younger than 18 years, of whom the 6 aged 6 years or younger were assessed with the CRS-P (11); the model has not been validated in children. Fifth, the category recorded as coma at the 6-month assessment should not be read as physiological coma. Coma is defined by loss of spontaneous arousal with sustained eye closure (31), and after acquired brain injury it seldom persists for more than three weeks in patients who survive; in survivors who recover wakefulness but continue to show no reproducible evidence of awareness, the appropriate diagnostic category is the vegetative state or the unresponsive wakefulness syndrome (32). Coma is retained here because it is the label that the registry uses for the lowest of the five levels, and the same caveat applies to the 39 patients recorded in that category at 1 month. None of the 15 patients with a 6-month category of coma was documented to have met the criteria for the minimally conscious state at any assessment, and the CRS-R total score, recorded at all three assessments in all 15, remained within the 0–3 range throughout; at 6 months, 10 were recorded as GOS 1 (death) and 5 as GOS 2 (vegetative state). In the five survivors, a bedside behavioral examination can be confounded by sedative or analgesic drugs, intercurrent illness, impaired vision or hearing, and limitation of motor or ocular movement, each of which can conceal residual awareness; the same consideration applies to any other category that is established from behavior alone. Because the primary outcome is the dichotomous distinction between achieving and not achieving eMCS, and none of these patients ever met the eMCS criteria, this uncertainty concerns the descriptive distribution of the patients who did not recover rather than the outcome definition: recoding the 15 patients to the vegetative state category leaves the analysis unchanged, excluding them gives an AUC of 0.76 (95% CI 0.70–0.82), and even if all 15 were assumed to have achieved eMCS the AUC of the multimodal model would be 0.73 (95% CI 0.67–0.80) rather than 0.77.

Future work should include multicenter prospective cohort studies with a bidirectional cohort design to validate and optimize the model. Subsequent studies could integrate functional MRI data and serum neuroinjury biomarkers such as neuron-specific enolase and S100B protein. More comprehensive multimodal prediction models using deep learning or other machine learning algorithms may further improve predictive performance. In addition, future studies could use pre-post treatment controlled designs to evaluate the relationship between stimulation parameters (frequency, intensity, duration) and consciousness improvement measured by CRS-R scores and neurobehavioral indicators. Such work would help establish treatment-response prediction models for neuromodulation interventions like spinal cord stimulation.

## Conclusion

Acute-phase FOUR score, age, etiology and the Fz P300 amplitude are independently associated with emergence from the minimally conscious state at 6 months in patients with prolonged DoC. A model combining nine acute-phase clinical and electrophysiological indicators discriminated moderately well between patients who did and did not reach eMCS (AUC = 0.772; bootstrap-corrected AUC 0.735), was adequately calibrated and modestly improved risk classification relative to clinical variables alone (continuous NRI = 0.317; IDI = 0.039). No significant difference in performance was detected between traumatic and non-traumatic etiologies, but the subgroup analyses were underpowered. The model requires external validation and prospective evaluation before it is used to inform clinical decisions; it is intended to complement, not replace, repeated clinical assessment.

## Data Availability

The raw data supporting the conclusions of this article will be made available by the authors, without undue reservation.
