# Supplementary material for: Prediction of emergence from the minimally conscious state at 6 months using acute-phase clinical and event-related potential indicators in patients with prolonged disorders of consciousness: a single-center retrospective cohort study
Source: Front Neurol. 2026 Oct 28;17:1948269. doi: 10.3389/fneur.2026.1948269 (PMC13751380; doi:10.3389/fneur.2026.1948269)
Supplement: Supplementary file 1 [file Table_1.docx]

# Supplementary Tables

Table S1. Variables and coefficients of the clinical, electrophysiological and multimodal models

| Model | Predictor | Beta (SE) | OR | 95% CI | P |
| --- | --- | --- | --- | --- | --- |
| Clinical model (6 variables) | Intercept | 0.568 (0.697) | 1.765 | 0.451-6.913 | 0.415 |
| Clinical model (6 variables) | Age, per year | -0.030 (0.011) | 0.970 | 0.949-0.992 | 0.008 |
| Clinical model (6 variables) | FOUR score, per point | 0.155 (0.048) | 1.168 | 1.062-1.284 | 0.001 |
| Clinical model (6 variables) | Etiology, TBI vs non-TBI | 0.970 (0.301) | 2.637 | 1.462-4.758 | 0.001 |
| Clinical model (6 variables) | Cerebral herniation, yes vs no | -0.588 (0.323) | 0.556 | 0.295-1.046 | 0.069 |
| Clinical model (6 variables) | Hydrocephalus, yes vs no | -0.256 (0.370) | 0.774 | 0.374-1.600 | 0.490 |
| Clinical model (6 variables) | st-SCS surgery, yes vs no | -0.977 (0.563) | 0.376 | 0.125-1.136 | 0.083 |
| Electrophysiological model (3 variables) | Intercept | -0.867 (0.286) | 0.420 | 0.240-0.736 | 0.002 |
| Electrophysiological model (3 variables) | Fz MMN amplitude, per uV | -0.182 (0.143) | 0.834 | 0.630-1.103 | 0.203 |
| Electrophysiological model (3 variables) | Cz MMN amplitude, per uV | -0.064 (0.153) | 0.938 | 0.695-1.266 | 0.677 |
| Electrophysiological model (3 variables) | Fz P300 amplitude, per uV | 0.137 (0.052) | 1.147 | 1.036-1.269 | 0.008 |
| Multimodal model (9 variables) | Intercept | -0.203 (0.777) | 0.816 | 0.178-3.746 | 0.794 |
| Multimodal model (9 variables) | Age, per year | -0.032 (0.012) | 0.969 | 0.947-0.992 | 0.007 |
| Multimodal model (9 variables) | FOUR score, per point | 0.174 (0.051) | 1.190 | 1.076-1.316 | <0.001 |
| Multimodal model (9 variables) | Etiology, TBI vs non-TBI | 0.762 (0.316) | 2.142 | 1.154-3.976 | 0.016 |
| Multimodal model (9 variables) | Fz MMN amplitude, per uV | -0.208 (0.168) | 0.812 | 0.584-1.129 | 0.216 |
| Multimodal model (9 variables) | Cz MMN amplitude, per uV | 0.032 (0.174) | 1.032 | 0.734-1.450 | 0.856 |
| Multimodal model (9 variables) | Fz P300 amplitude, per uV | 0.146 (0.058) | 1.157 | 1.033-1.295 | 0.012 |
| Multimodal model (9 variables) | Cerebral herniation, yes vs no | -0.488 (0.333) | 0.614 | 0.319-1.180 | 0.143 |
| Multimodal model (9 variables) | Hydrocephalus, yes vs no | -0.448 (0.385) | 0.639 | 0.300-1.359 | 0.244 |
| Multimodal model (9 variables) | st-SCS surgery, yes vs no | -0.857 (0.573) | 0.424 | 0.138-1.304 | 0.134 |

Table S2. Internal validation, calibration and goodness-of-fit of the three models

| Model | AUC (95% CI) | Optimism | Bootstrap-corrected AUC | 10-fold cross-validated AUC | Calibration slope (apparent) | Calibration slope (bootstrap-corrected, 95% CI) | Brier score | Scaled Brier score (R2 analogue) | Hosmer-Lemeshow chi-square (df) | H-L P value |
| --- | --- | --- | --- | --- | --- | --- | --- | --- | --- | --- |
| Clinical (6 variables) | 0.745 (0.681-0.809) | 0.025 | 0.720 | 0.711 | 1.00 | 0.86 (0.59-1.19) | 0.204 | 0.184 | 5.45 (8) | 0.708 |
| Electrophysiological (3 variables) | 0.654 (0.583-0.726) | 0.016 | 0.638 | 0.621 | 1.00 | 0.90 (0.55-1.48) | 0.232 | 0.069 | 10.11 (8) | 0.257 |
| Multimodal (9 variables) | 0.772 (0.711-0.832) | 0.037 | 0.735 | 0.730 | 1.00 | 0.80 (0.57-1.08) | 0.194 | 0.223 | 6.12 (8) | 0.634 |

Table S3. Sensitivity analyses

| Analysis | n | Events | AUC (95% CI) | Note |
| --- | --- | --- | --- | --- |
| Primary analysis (all patients) | 227 | 118 | 0.772 (0.711-0.832) |  |
| Adjusted for interval from onset to ERP | 227 | 118 | 0.771 (0.711-0.832) | OR per day 1.014, P = 0.541 |
| Interval from onset to ERP alone | 227 | 118 | 0.481 (0.405-0.557) | sensitivity model, not a prediction model |
| ERP within 28 days of onset | 208 | 111 | 0.775 (0.712-0.838) |  |
| ERP 2-14 days after onset | 71 | 37 | 0.892 (0.819-0.965) |  |
| ERP 15-31 days after onset | 156 | 81 | 0.750 (0.674-0.826) |  |
| Adults only (age >= 18 years) | 218 | 110 | 0.762 (0.699-0.825) |  |
| Excluding patients who died (GOS 1) | 181 | 117 | 0.787 (0.719-0.855) |  |
| Excluding ERP recorded before day 7 | 216 | 113 | 0.767 (0.704-0.829) |  |
| Excluding patients already in eMCS at 1 month | 199 | 90 | 0.722 (0.652-0.793) | prediction restricted to patients in VS or MCS at 1 month |
| Excluding patients who had reached eMCS at 3 months | 131 | 22 | 0.732 (0.601-0.863) | additional check of reverse causation |
| Alternative outcome: GOS >= 3 at 6 months | 227 | 125 | 0.765 (0.704-0.826) | outcome redefined, predictors unchanged |
| Alternative outcome: CRS-R total >= 20 at 6 months | 227 | 113 | 0.760 (0.698-0.823) | outcome redefined, predictors unchanged; 5 patients (2.2%) reclassified relative to the eMCS definition |
| Alternative outcome: CRS-R total >= 22 at 6 months | 227 | 96 | 0.754 (0.689-0.819) | outcome redefined, predictors unchanged; 22 patients (9.7%) reclassified relative to the eMCS definition |
| Alternative outcome: CRS-R total >= 23 at 6 months | 227 | 81 | 0.753 (0.686-0.819) | outcome redefined, predictors unchanged; 37 patients (16.3%) reclassified relative to the eMCS definition |
| Complete-case analysis (complete ERP data) | 220 | 114 | 0.771 (0.709-0.833) | 7 patients with missing ERP values excluded |
| Backward elimination at P < 0.05 (from the nine candidates) | 227 | 118 | 0.753 (0.691-0.816) | retained age, FOUR score, etiology, cerebral herniation and Fz_P300_A; forward selection retained the same five variables; DeLong test against the pre-specified nine-variable model P = 0.196 |
| Backward elimination by AIC (from the nine candidates) | 227 | 118 | 0.768 (0.707-0.829) | retained seven variables; DeLong test against the pre-specified nine-variable model P = 0.564 |

Table S4. Subgroup analyses

| Stratum | n | Events | AUC (95% CI) | P for interaction vs the other stratum |
| --- | --- | --- | --- | --- |
| Etiology: TBI | 122 | 79 | 0.794 (0.714-0.873) | 0.346 |
| Etiology: non-TBI | 105 | 39 | 0.733 (0.635-0.831) | - |
| Likelihood-ratio test for all predictors x etiology interactions | 227 | 118 | - | 0.271 |
| Age below the median (59 years) | 121 | 64 | 0.796 (0.717-0.875) | - |
| Age at or above the median (59 years) | 106 | 54 | 0.818 (0.739-0.897) | - |
| FOUR score below the median (6) | 114 | 50 | 0.800 (0.720-0.880) | - |
| FOUR score at or above the median (6) | 113 | 68 | 0.798 (0.712-0.883) | NA |

Table S5. Risk reclassification (NRI and IDI) of the electrophysiological and multimodal models relative to the clinical model

| Comparison | Continuous NRI | Continuous NRI 95% CI | P | IDI | IDI 95% CI | P | Categorical NRI (0.2/0.5) | Categorical NRI 95% CI | P |
| --- | --- | --- | --- | --- | --- | --- | --- | --- | --- |
| Multimodal (9 variables) vs clinical (6 variables) | 0.317 | 0.062-0.572 | 0.015 | 0.0395 | 0.0137-0.0652 | 0.003 | 0.056 | -0.055-0.167 | 0.325 |
| Electrophysiological (3 variables) vs clinical (6 variables) | -0.540 | -0.790--0.290 | <0.001 | -0.1135 | -0.1711--0.0560 | <0.001 | -0.240 | -0.416--0.064 | 0.008 |

Table S6. Consciousness and functional outcome at 6 months

| Item | Overall | Not_achieved | Achieved | P_value |
| --- | --- | --- | --- | --- |
| CRS-R total score at 6 months, median (IQR) | 19 (8-23) | 8 (4-12) | 23 (22-23) | <0.001 |
| CRS-R total score < 23 at 6 months among patients who achieved eMCS, n (%) | - | - | 37 (31.4) | - |
| Glasgow Outcome Scale at 6 months, n (%) |  |  |  | <0.001 |
| 1 (death) | 46 (20.3) | 45 (41.3) | 1 (0.8) |  |
| 2 (vegetative state) | 56 (24.7) | 55 (50.5) | 1 (0.8) |  |
| 3 (severe disability, dependent) | 66 (29.1) | 9 (8.3) | 57 (48.3) |  |
| 4 (moderate disability, independent) | 19 (8.4) | 0 (0.0) | 19 (16.1) |  |
| 5 (good recovery) | 40 (17.6) | 0 (0.0) | 40 (33.9) |  |
